# The Horseshoe Crab of the Genus *Limulus*: Living Fossil or Stabilomorph?

**DOI:** 10.1371/journal.pone.0108036

**Published:** 2014-10-02

**Authors:** Adrian Kin, Błażej Błażejowski

**Affiliations:** 1 Society of Friends of Earth Sciences *PHACOPS*, Warsaw, Poland; 2 Institute of Paleobiology, Polish Academy of Sciences, Warsaw, Poland; Monash University, Australia

## Abstract

A new horseshoe crab species, *Limulus darwini*, is described from the uppermost Jurassic (ca. 148 Ma) near-shore sediments of the Kcynia Formation, central Poland. The only extant species *Limulus polyphemus* (Linnaeus) inhabits brackish-marine, shallow water environments of the east coast of the United States. Here it is shown that there are no important morphological differences between the Kcynia Formation specimens and extant juvenile representatives of the genus *Limulus*. The palaeoecological setting inhabited by the new species and the trophic relationships of extant horseshoe crabs are discussed in an attempt to determine the potential range of food items ingested by these Mesozoic xiphosurans. In this paper we propose the adoption of a new term stabilomorphism, this being: an effect of a specific formula of adaptative strategy among organisms whose taxonomic status does not exceed genus-level. A high effectiveness of adaptation significantly reduces the need for differentiated phenotypic variants in response to environmental changes and provides for long-term evolutionary success.

## Introduction

**Figure 1:**
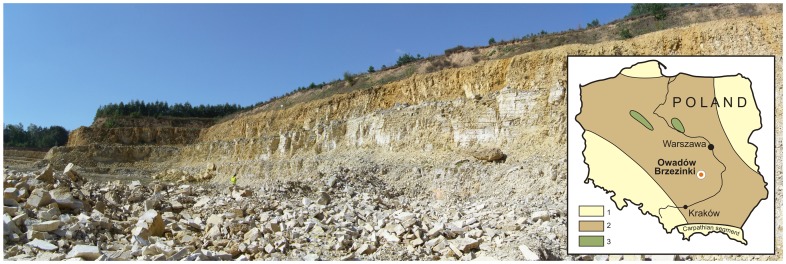
Panoramic view of the highest level of exploitation in Owadów-Brzezinki Quarry (i.e. unit III and most fossiliferous ‘*Corbulomima* horizon’ occurring in the middle of the quarry wall) and locality map. Key: 1, Marine sediments, not studied in detail. 2, Shallow water limestone. 3, Siliclastic, fine grained sediments.

**Figure 2:**
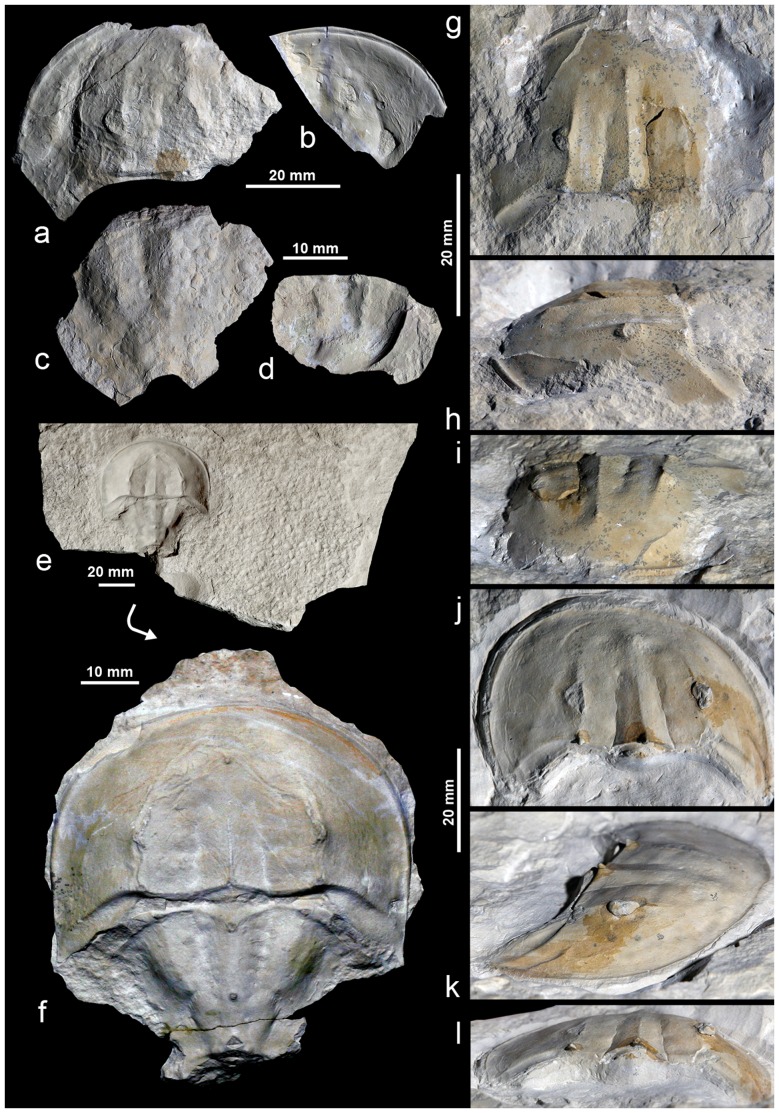
Three-dimensionally preserved representatives of Late Jurassic *Limulus darwini* that can be assigned to three major size classes. (**a**) compressed and incomplete prosoma (ZPAL X.1/O-B/XA 5); (**b**) Markedly incomplete prosoma (ZPAL X.1/O-B/XA 4); (**c**) slightly incomplete opisthosoma (ZPAL X.1/O-B/XA 6); (**d**) small, incomplete opisthosoma (ZPAL X.1/O-B/XA 7); (**e**), (**f**) Positive (rock slab with imprint) and negative (external mould) of the holotype (ZPAL X.1/O-B/XA 1), preserved as slightly compressed and juxtapositioned prosoma and opisthosoma; (**g**–**i**) least-deformed specimen (ZPAL X.1/O-B/XA 2), preserved as a near-complete, medium-sized prosoma; (**j**–**l**) flattened, complete prosoma (ZPAL X.1/O-B/XA 3), with exceptionally well-preserved right compound eye.

**Figure 3:**
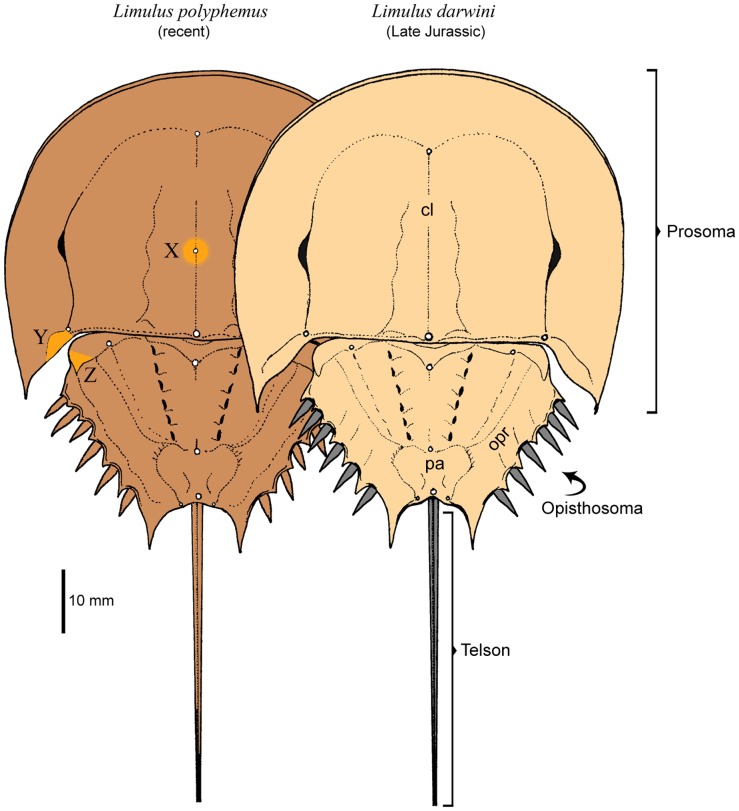
Comparison of modern *Limulus polyphemus* (left) and oldest known member of the genus *Limulus darwini* (right) from *Corbulomima* horizon of unit III from Late Jurassic (upper Tithonian = Middle Volgian) sedimentary sequence at Owadów-Brzezinki Quarry (central Poland). (X), (Y) and (Z) - details emphasized, are most substantial morphological difference between both these forms. (cl) - cardiac lobe; (opr) – opisthosomal rim; (pa) – posterial area. Morphological elements of *L. darwini* exoskeleton not known from the fossil record (i.e. movable spines and telson) emphasized in grey.

Xiphosurida arthropods are amongst the rarest of macrofossils. Thus the discovery of new, three-dimensionally preserved Late Jurassic Xiphosurida material adds significantly to our understanding of a group, the stratigraphic range of which spans almost the entire Phanerozoic. The aim of this paper is to describe a new species, *Limulus darwini*, from the Late Tithonian Kcynia Formation at the Owadów-Brzezinki Quarry, near Tomaszów Mazowiecki in central Poland (Figure 1). Limestones of this unit have yielded a total of eight well-preserved specimens, including one almost completely articulated exoskeleton. The other seven are incomplete; these are preserved as isolated fragments of the prosoma and opisthosoma (Figure 2, for anatomy see Figure 3). Specimens of *L. darwini* sp. nov. constitute the first Late Jurassic representatives of the order Xiphosurida to be recorded from Poland. So far, the sole limulid on record was a specimen of *Limulitella* cf. *liasokeuperinus* [1] from a brackish-marine horizon in the lower Pliensbachian Gielniów Formation at Skarżysko-Kamienna (i.e. south-central Poland – see [2], [3]); however, this particular specimen was lost during World War II. Mesozoic specimens of horseshoe crabs were extremely rarely found in the fossil record. Previous studies of Xiphosurida of the families Paleolimulidae, Mesolimulidae, and Limulidae of the Limulacea (*sensu* [4]) have revealed mainly time-related, distinguishing features which have been based on often inconsistent phylogenetic and taxonomic criteria. Complicating matters further is the fact that extinct horseshoe crabs are extremely rare and that their remains are usually imperfectly preserved. Detailed analyses of the phylogenetic relationships between extinct and extant families and genera are therefore very difficult [5]. A perusal of the literature reveals that, almost every new find is given the rank of a new genus [6]. Even the proposal by Riek and Gill [7] to place the genus *Mesolimulus* [4] in the family Limulidae thereby eliminating the family Mesolimulidae [4] (considered here to be valid), does not significantly alter this situation.

After a detailed analysis of three-dimensionally preserved Late Jurassic limulids from Owadów-Brzezinki, it should be explicitly stated that there are no significant morphological differences between these and extant juvenile individuals of the genus *Limulus* [8] (Figure 3). Without much doubt modern horseshoe crabs of that genus probably are even closer to Late Jurassic forms than previously thought [5], [7]. The morphological features of the opisthosoma of these are very similar to the holotype of *Limulus coffini* [9], a perfectly preserved, non-flattened opisthosoma from the Upper Cretaceous of Colorado, USA. Presumably both forms were closely related, while *L. darwini* most probably constitutes an early link both with *L. coffini* and the extant *Limulus polyphemus* [8]. This also means that the genus *Limulus* existed about 148 million years ago and has survived to the present day in an almost unchanged form. Accordingly, *L. darwini* is here regarded as the oldest known representative of the genus. However, the phylogenetic relationship of the new form with the Early Cretaceous *Victalimulus mcqueeni* [7] of Australia and *Crenatolimulus paluxyensis* [6] of North America cannot be determined fully until more better preserved specimens of the latter are found.

### Geological and palaeontological settings

The study area is located about 18 km southeast of Tomaszów Mazowiecki (central Poland; for details, see [10]). Here Late Tithonian ( = Middle Volgian) carbonate sediments are exposed in a small working quarry belonging to the Nordkalk Company (Sławno). At the moment, the Owadów-Brzezinki Quarry is the only place in extra-Carpathian Poland where the Upper Tithonian strata are available for study (the classic locality Brzostówka is now within the Tomaszów Mazowiecki town limits; quarries in Pomerania are flooded). The exposed carbonate sequence belongs to the Kcynia Formation, and can be divided into four successive units. Unit I is composed of indistinctly laminated massive fine-grained limestone (∼6.6 m total thickness) with *Deltoideum delta*, which forms a few beds of 40–80 cm thickness. The overlying unit II, c. 2 m thick, is represented by thinly-bedded, fine-grained limestones with occasional distinctive parallel lamination and mass occurrence of calcareous polychaete tubes in one horizon. Unit III, c. 15.6 m thick, is highly fossiliferous and has yielded the horseshoe crabs that are the subject of the research reported here. Unit IV, c. 2.3 m thick, the top of which is unexposed, is developed as organodetrital limestone rich in *Nanogyra* oysters, bryozoans and serpulids. They often form small bioherms. In general, units I, II and III probably represent a transition from an offshore to nearshore, perhaps lagoonal, setting, whereas Unit IV bears evidence of a return to more open marine conditions. Yellowish marls and marly clays of the Pałuki Formation occur lay below the Kcynia Formation.

The uppermost part of the unit (III) is highly fossiliferous, with a horizon of finely bedded fine-grained limestones at its base (also called the ‘*Corbulomima* horizon’), dominated by the small opportunistic bivalve *Corbulomima obscura* and somewhat less numerous *Mesosaccella* sp. It follows that the *Corbulomima* horizon in the higher portion of the section (i.e. unit III), from which all specimens of *L. darwini* originate, was laid down in a very shallow marine basin (perhaps lagoon), which had rather limited links with the open sea [11]. The constant proximity of the open sea is indicated by occasional finds of ammonites of the genus *Zaraiskites* [12].

In the unit III representatives of the opportunistic soft-shelled bivalve genus *Corbulomima* [13], disarticulated remnants of various marine and land arthropods (including decapods, beetles, dragonflies [14] and grasshoppers) and moulds of ammonite shells are commonly associated with *L. darwini* sp. nov.

The vertebrate fauna comprises disarticulated fish remains (e.g. post-cranial skeleton of pycnodontiform fish), sphenodonts and a few skeletal remains of small indeterminate rhamphorhynchid pterosaurs [11].

Clearly there are many interesting parallels between the rocks of Owadów-Brzezinki Quarry and the classic Solnhofen lithographic limestone [11]. The fossils identified at both localities indicate a similar geological age and environment. Both marine and terrestrial organisms are very similar and allow comparative palaeontological studies at a previously unattainable level of taxonomic resolution. The recent identification of a new species of dragonfly (family Eumorbaeschnidae) and grasshopper (family Prophalangopsidae), families previously known only from the Solnhofen area, is an example. Clearly, the Owadów-Brzezinki Quarry may be regarded as a new ‘taphonomic window’ into the living world of the latest Jurassic and it represents the first near-contemporary palaeontological ‘supplement’ to Solnhofen (lower Tithonian) and Nussplingen (uppermost Kimmeridgian). The small palaeochronological distance (5–7 Ma) separating these three locations may enable us to trace lineages, with different stages of evolutionary advancement recorded by the fossils from these localities.

### Preservation of the studied horseshoe crabs

Limulid specimens in this study represent more or less three-dimensionally preserved elements of exuvia representing different age groups and occurring in three major size classes (Table 1). The holotype of *L. darwini* (ZPAL X.1/O-B/XA 1; Figure 2e, f; Table 1) is preserved as a slightly compressed and juxtapositioned complete prosoma and near-complete opisthosoma, but lacks opisthosomal spines. The absence of the telson can presumably be explained by exuvial disarticulation (i.e. stage 3 *sensu* [15]). One of the best preserved specimen, ZPAL X.1/O-B/XA 2 (Figure 2g–i; Table 1), is a near-complete, medium-sized prosoma. Specimen ZPAL X.1/O-B/XA 3 (Figure 2j–l; Table 1) is preserved as a slightly flattened, complete prosoma, with exceptionally well-preserved right compound eye. Another specimen (ZPAL X.1/O-B/XA 5; Figure 2a), not included in the biometric analysis, is preserved as a flattened, incomplete prosoma, with an estimated length/width of c. 55 mm/82 mm. ZPAL X.1/O-B/XA 6 (Figure 2c; Table 1) is a slightly incomplete opisthosoma. The two last-named specimens are parts of a single disarticulated moult (both specimens were found close to each other on the same surface stratum). Thus the maximum size of the new species must have been approximately 105 mm (without telson). Specimen ZPAL X.1/O-B/XA 4 (Figure 2b), also not included in the biometric analysis, is preserved as an incomplete prosoma (size, as preserved, 47 mm by 58 mm); its original length and width may be estimated at 53 mm and 80 mm, respectively. The two smallest specimens available are comparable in size, and are both preserved as incomplete opisthosomas: ZPAL X.1/O-B/XA 7 (Figure 2d; Table 1) and ZPAL X.1/O-B/XA 8 (not illustrated).

**Table 1:** Table of biometric measurements of selected specimens *Limulus darwini* sp. nov.

*Limulus darwini* sp. nov.					
Specimens	ZPAL X.1/O-B/XA 1	ZPAL X.1/O-B/XA 2	ZPAL X.1/O-B/XA 3	ZPAL X.1/O-B/XA 6	ZPAL X.1/O-B/XA 7
**Prosomal length**	34.3 mm	33 mm	29 mm	-	-
**Prosomal width**	63 mm	∼52 mm	56 mm	-	-
**Cardiac lobe length**	14.2 mm	20 mm	15.5 mm	-	-
**Cardiac lobe width**	10.2 mm	10 mm	11 mm	-	-
**Interocular distance**	29 mm	22 mm	22 mm	-	-
**Opisthosomal length**	31.8 mm	-	-	55 mm	∼16 mm
**Opisthosomal width**	26 mm	-	-	38 mm	∼22 mm
**Axial width**	12.2 mm	-	-	∼19 mm	7 mm
**Last marginal spine length (anal angle)**	8.3 mm	-	-	∼11 mm	-
**Opisthosomal rim width**	8.6 mm	-	-	∼11 mm	∼3.8 mm
**Posterial area length**	11 mm	-	-	18 mm	∼7 mm
**Posterial area width**	15.3 mm	-	-	24 mm	∼9 mm
**Total length of prosoma & opisthosoma**	66.8 mm	-	-	-	-

## Results

### Systematic palaeontology

Phylum ARTHROPODA [16]

Order XIPHOSURIDA [17]

Family LIMULIDAE [18]

Genus *Limulus* [8]

***Type species*** *. Limulus polyphemus* Linnaeus, C. 1758. Systema Naturae. Ed. 10(1), 1–824. Salvii, Holmiae

*Limulus darwini* sp. nov.

***Diagnosis***.–Smaller than the type species, only up to 15% the size (although entirely represented by non-fully grown specimens, possible juveniles); absence of a median cardiac node (which is normally present in the type species); relatively long, narrow and moderately depressed shape of the occipital bands (instead of short, wide and shallow lobes in the type species); smaller size and more slender articular processes (instead of considerably larger and posteriorly expanded in the type species).

*Limulus darwini* Kin & Błażejowski 2014 sp. nov. urn:lsid:zoobank.org:act:FC04D675-4D90-4A32-8C1F-CC0FD04B0A19

***Derivation of name***.–In honour of Charles Robert Darwin (1809–1882), for his invaluable contributions to our knowledge of evolution and natural history.

***Type locality***.–Owadów-Brzezinki Quarry, close to Tomaszów Mazowiecki, central Poland, 51°22′34.53″N 20°08′07.86″E.

***Holotype***.–ZPAL X.1/O-B/XA 1 (Figure 2 e, f), stored in the collections of the Institute of Paleobiology, Polish Academy of Science in Warsaw (ZPAL X.1/O-B).

***Other material***.–Eight specimens (paratypes), ZPAL X.1/O-B/XA 2 to XA 9.

***Stratigraphical range***.–*Corbulomima* horizon, Kcynia Formation, of Late Tithonian (Middle Volgian - *Scythicus* Zone, *Zarajskensis* Subzone and Horizon of Kutek [19]) age.

***Geographical range***.–To date, known from the type locality only.

***Measurements***.–See Table 1.

*Description*.–The holotype has a semicircular prosoma which is approximately 1.5 times wider than long. Its dorsal surface is delimited by a clear and uniformly narrow marginal rim. A single pair of relatively large compound eyes is situated on the well-developed ophthalmic ridges, posterior to the prosoma. The ophthalmic ridges terminate as two tubercles on the posterior part of the prosoma, and do not meet in front of the cardiac lobe. The interophthalmic region is bordered by two rows of poorly marked muscular impressions. The preophthalmic field is moderately wide, with a centrally positioned ocellus at its base. The sharply defined cardiac ridge forms a small median tubercle (in the posterior part) and becomes weaker halfway between the median tubercle and the ocelli. The genal spines are short; their outer margins almost parallel with the median axis of the body; the occipital bands are relatively narrow.

The opisthosoma is nearly hexagonal in outline and consists of a single sclerite with a poorly distinguished axis, without any transverse annulations or longitudinal ridge. The larger anterior part of opisthosoma consists of a small microtergite and six pairs of distinct apodomes ( = entapophyseal pits). The abdominal axis has two axial tubercles, the first halfway between the primary and secondary pairs of apodomes, the second slightly below the border (i.e. large muscle scars) with the posterior part of opisthosoma. The surface of the smaller posterior opisthosomal part, whose axis consists of two segments, forms a subhexagonal area and is separated by a posteriorly elongated and tapering area with three tubercles. The first is a centrally embedded posterior axial tubercle. The other two tubercles are much smaller and are symmetrical, on either side of the posterior axial tubercle. On the anterior ridges there are two symmetrically positioned tubercles. In the region of the first abdominal segment, closer to the axis, are two small articular processes. The moderately wide opisthosomal rim consists of six pairs of short and symmetrically arranged spines, and is terminated posteriorly by two large marginal spines. The structure of the venter (e.g. appendages, genital operculum, flabellum etc.) and construction of movable spines and telson are unknown.

### Comparison of *Limulus darwini* with extinct and extant limulines

In broad terms, amongst all known extinct Mesozoic and Cenozoic horseshoe crabs (for details [4], [6], [20]) *Limulus darwini* shows general similarities almost identical with the well-known Late Jurassic limuline from Germany and two exceptionally preserved early Cretaceous limulines from USA (details below). This also means that other extinct post-Palaeozoic horseshoe crabs were not taken into account in comparison presented here *inter alia* because of lack of sufficiently well-preserved palaeontological materials (e.g. *Victalimulus mcqueeni* – see above) or recognizable morphological dissimilarity in relation with *L. darwini* (e.g. Middle Triassic *Yunnanolimulus luopingensis* [21]). Xiphosurans have existed for some 480 Mya [22], with the earliest unequivocal representatives found from the Upper Ordovician of Manitoba, Canada [23], followed by further Xiphosurida reports from the Lower Ordovician of Morocco [24].

The new species described in this article shows some features in common with representatives of the genus *Mesolimulus*, in particular with *M. walchi* [25] from the late Kimmeridgian pelitic limestones of Brunn [26] and Nusplingen [27], [28], and the early Tithonian lithographic limestones of Solnhofen [29], [30]. *Limulus darwini* differs from *M. walchi* mainly in having a narrower furrow along the inner margin of the genal angles on the prosoma and in the presence of short articular processes, plus the absence of distinct dorsal furrows on the opisthosoma. Among other features which distinguish these two species are the slender shape of the opisthosoma, the absence of a longitudinal ridge on the axis and the distinct separation of the posterior abdominal sclerite in the new species. Unfortunately, on the basis of material available from Germany, it cannot be determined unambiguously whether or not the last-mentioned differences are a function of the state of preservation of the *L. darwini* specimens, which are significantly less compressed.

The holotype of *C. paluxyensis*, from the Lower Crataceous (Albian) Glen Rose Formation in north-central Texas, has two special morphological features that distinguish it from all other post-Palaeozoic horseshoe crabs i.e. the beaded rims of the posterior region on the prosoma and flanks with two prominent ridges defining a tripartite surface on the opisthosoma [6]. The other exoskeleton features can be considered as generally similar to *L. darwini*, with the special exception of the proportionally much longer genal spines on the prosoma and clearly pronounced axial ridge on the opisthosoma. It is well understood that, during ontogenetic development, the modern horseshoe crabs follow the gradual change in proportions of the prosoma and opisthosoma as well as in dorsal sculpture [31]–[33]. Therefore, in our opinion, the significantly larger size of the holotype *C. paluxyensis* (length/wide diameters: 234 mm and 296 mm) precludes a detailed morphological comparison with relatively small exuvia belonging to the new species described herein (see above, Table 1).

Comparisons may be made with another extinct horseshoe crab *Limulus coffini*, which is preserved as a three-dimensionally complete opisthosoma (length c. 70 mm). This specimen shows similar morphological characteristics with the abdominal parts of *L. darwini*. Among the most important are six fixed spines terminated posteriorly by two large marginal spines occurring on both side of the relatively deep and wide posterior margin, six distinct apodomes on either side of the axis, and large muscle scars separating anterior and posterior opisthosomal parts. Among morphological features that distinguish *L. darwini* from *L. coffini* are: the lack of a pronounced axial ridge, the occurrence of three (not two) tubercles along the axial ridge and somewhat larger articular processes for the new species from Poland, although it should be noted that the spines on recent *Limulus* are quite variable even within a population.

In comparably sized individuals, i.e. juveniles, of *L. polyphemus* (compare [15]), the separation of the larger anterior part and abdominal posterior part of the opisthosoma in *L. darwini* closely resembles the same opisthosomal area. Other morphological features of *L. darwini* also reveal a marked similarity to juvenile representatives of the genus *Limulus*; e.g. the degree of curvature and the general outline of the external margin of the genal lobe, the presence of moderately large lateral eyes placed posteriorly on the prosoma, cardiac lobes and cheek areas which have comparable shape and size proportions, the absence of a clearly marked opisthosomal axis, the presence of six pairs of distinct entapophyseal pits, the position of the first pair of marginal spines, the occurrence of three median tubercles along the axis of the opisthosoma, comparatively deep and wide posterior opisthosomal margin, and marginal spines with closely related outline and size. The similarities between *L. darwini* and *L. polyphemus* listed above, refer only to the dorsal part of their exoskeletons and, at the present time, it is only possible to carry out a limited comparison in this regard. However, a very high level of external parallels of the dorsal parts of both species (reaching about 85% of corresponding features - Figure 3) is highly suggestive of a relationship at genus level. The features noted distinguish the dorsal part of the exoskeletons of both forms, as illustrated in Figure 3. The most important of these are the presence of a median cardiac node (about halfway down the length of the cardiac rim, marked X) and the shape of the occipital bands (marked Y) on the prosoma, and also the size and shape of the articular processes (marked Z) on the opisthosoma.

Otherwise, with regard to similar sized (juvenile) horseshoe crabs belonging to the other two extant taxa – *Tachypleus* [34] and *Carcinoscorpius* [35] – *L. darwini* shows numerous important morphological differences. Among selected examples from both recent juvenile forms are: the compound eyes of *L. darwini* are positioned more anteriorly and their intraophthalmic distance is considerably smaller, the cheek area is much larger and more extensive (especially in the lateral and posterior direction), the cardiac lobes become narrower anteriorly and form a sub-triangle area with a more or less sharp outline, articular processes have different shapes and are directed entirely toward the outside (i.e. forming distinct angle with the opisthosomal axis).

Additionally, in *Carcinoscorpius* the genal spines are considerably broader and the shape of the opisthosoma is regularly hexagonal with a much wider opisthosomal rim. The general similarity of the dorsal part of the exoskeletons of the three above-mentioned genera achieves only about 55–65% [36]; however, this also seems to be a common similarity level between particular taxa of known (both extinct and extant) representatives of the family Limulidae (for details, see [6]). A recent comparison of the modern American versus the Asiatic horseshoe crabs genera, based upon molecular patterns, shows that *Limulus* is a sister taxon to the other two genera [37].

In the presently recovered specimens, those of a size comparable to mature individuals of *L. polyphemus* are lacking, and the absence of fully-grown exoskeletons of late Jurassic horseshoe crabs from the fossil record remains enigmatic. According to preliminary assumptions made by Barthel [31], all specimens of *M. walchi* available were probably exoskeletons (moults) of young animals. Lending support to this observation are the finds of giant limulid trackways (i.e. *Kouphichnium lithographicum* [38] in the Upper Jurassic of Germany [30], [39], [40] and France [41]) which have been attributed to much larger (adult?) specimens. We suggest that the absence of large moults of *M. walchi* and *L. darwini* may, in part, have been caused by the much rarer moulting amongst subadult horseshoe crabs (usually once per year), in comparison to juveniles (up to several times), as has been documented for modern limulids [31]–[33], [42].

Thus, it is possible that this was the same in *L. darwini*. The occurrence of moults of three age groups in *L. darwini* in the shallow-water sediments at Owadów-Brzezinki may thus be an example of a limulid nursery. In extant *L. polyphemus* and *T. tridentatus*, nursery grounds are found in shallow coastal zones, where young horseshoe crabs spend the first years rapidly increasing its size, prior to venturing out into deeper waters [32], [33].

The close morphological similarity between *L. darwini* and juvenile specimens of *L. polyphemus* is very apparent (Figure 3). The prosomal width of small (juvenile) representatives of *Limulus* and *Tachypleus* usually does not exceed 29 mm; in older juveniles it is estimated to be between 29 mm and 168 mm [32], [42]. Based on these data, we contend that all specimens of *L. darwini* currently available pertain exclusively to moults of juveniles (compare Table 1).

In this respect, the absence of epibionts on small to moderate large exoskeletons of extinct forms, such as Late Jurassic *M. walchi* and *L. darwini*, is also significant. Among extant adult horseshoe crabs a commonly observed phenomenon is the presence of differentiated epibiont associations (e.g. barnacles, bryozoan and slipper shell); these may cover large regions of the carapaces of some individuals [43]. Juveniles rarely have epibionts owing to their active burying in the sediment and the presence of mucus secretions on the dorsal parts, which allow them to remove from their exoskeletons any fouling organisms. However, it should be emphasized that adult horseshoe crabs only occasionally exhibit this (burrowing) type of behavior, replacing it by roaming on the surface and shallow digging [31]. Possibly a more important reason is that juvenile horseshoe crabs molt frequently and would therefore not be able to provide a suitable substrate for the epibionts [44]. It seems to be important that the specimens collected so far from Owadów-Brzezinki locality have the potential for studying evolutional changes within the lineage in the timing of development (heterochrony) in which juvenile features are retained by the adult (paedomorphosis).

### Palaeoecology of the *Corbulomima* horizon at Owadów-Brzezinki

Environmental interpretations of limestones of the *Corbulomima* horizon (unit III) are controversial. In the near-monotypic faunal assemblages (97 per cent *Corbulomima* sp. exhibiting internal mould preservation – [11]), organisms that would indicate a clearly brackish-water setting are missing; in addition, there is a total lack of sedimentological indicators of hypersalinity (e.g. authigenic gypsum crystals). Specimens of *Corbulomima* sp. from unit III are characterised by a slight variation in shell shape and ornament, resembling what is known from the extant euryhaline bivalve *Potamocorbula amurensis* [45] from the western Pacific [46]. Observations based on modern members of the family Corbulidae [47], a group which first appeared in the Middle Jurassic and which includes about 35 nominal genera, 15 of them extant (see [48]–[50]) have demonstrated a high degree of tolerance to salinity fluctuations. The maximum level has been recorded for *Potamocorbula amurensis* and *Varicorbula gibba* [51], i.e. between 0 and 30 psu, and this remains almost constant at all growth stages [46], [52], [53]. Detailed studies of palaeoecological preferences of extinct corbulids have enabled similar conclusions to be reached [48], [54]–[57]. This means that at least some fossil corbulids must have been euryhaline and inhabited water of varying salinity. This, of course, limits their value for detailed palaeoecological analyses. Potentially, their mass occurrence might be suggestive only of episodic physical and/or chemical environmental stress [58].

### Diet of recent limulids

The food intake of modern Atlantic *Limulus polyphemus* is highly diverse and consists normally of bivalves, gastropods, polychaetes, crustaceans but also when available echinoids, campanularids, teleost fish, foraminifera and a small amount of plant material [59]–[61]. Similar dietary preferences have been identified in other extant species such as *Tachypleus tridentatus* [34], *Tachypleus gigas* [8] and *Carcinoscorpius rotundicaudata* [17], as demonstrated by Chatterjee et al. [62], [63] and Zhou and Morton [64]. A consistent element in the diet of all modern horseshoe crabs is organic detritus, consisting mainly of plant debris [63], [65]. It is certainly the case that the diet composition of modern representatives varies at different growth stages and is strictly dependent on seasonal food availability which, in turn, reflects the food sources in the inhabited geographical area.

Juvenile and adult *L. polyphemus* prey mainly on invertebrates; for juveniles, this includes bivalves of the genera *Mya*, *Ensis*, *Mulinia*, *Macoma*, *Spisula*, *Mytilus*, *Tellina*, *Gemma* and *Siliqua*, the worms *Nereis* and *Cerebratulus*, and the gastropod genera *Nassarius*, *Olivella*, *Polinices*, *Turbonilla*, *Odostomia* and naticids [60], [61], [65]–[70]. Indo-Pacific forms (*T. tridentatus*, *T. gigas* and *C. rotundicaudata*) feed on bivalves of the genera *Anadara*, *Dosinia*, *Placenta*, *Macoma*, *Solen*, *Neosolen*, *Teredo*, a variety of worms (e.g. *Gattyana*, *Phyllodoce*, *Nereis*, *Perinereis*) and gastropods of the genera *Cerithedia*, *Littorina*, *Assimenea*, *Nerita*, *Cassidula* and *Cymia* [71].

However, in terms of statistical representation, among all macrobenthic organisms the most favoured food items are plant debris and small, soft-shelled molluscs (i.e. bivalves and, to a lesser extent, snails). Furthermore, studies conducted on populations of modern *L. polyphemus*, which inhabits most of the eastern North American continental shelf (i.e. from Maine to Florida and parts of the Yucatan Peninsula in Mexico), have demonstrated that the dominant food component may almost exclusively be bivalves, which accounted for up to 87 per cent of total diet for horseshoe crabs examined [61].

## Discussion

### Potential feeding habits of *Limulus darwini* sp. nov

Owing to a paucity of data, the potential importance of bivalves in the diet of Mesozoic horseshoe crabs cannot yet be fully assessed. The obvious reason for this is the rarity of finds of limuloids of this age and the fact that, in general, there is no conclusive evidence whether an investigated area formed the primary setting in which they lived, or represented merely the area of final burial. Based on available data, only an estimate of the percentage and environmental character of the bivalves in macrofossil assemblages that have yielded Mesozoic Xiphosurida can be made. For example, in the upper Kimmeridgian of Nusplingen (southern Germany), where *Mesolimulus walchi* is very rare [28], bivalve fossils are uncommon and represent only an allochthonous element [72]. Thus, the Nusplingen area probably did not constitute an attractive feeding ground for *M. walchi*, but could have formed a potential trap for inexperienced or stressed individuals, as in the case of lethal lagoons of the Solnhofen and Cerin (France) areas [31], [41].

As discussed above, bivalves appear to be basic components of the diet of recent horseshoe crabs. These are represented by different taxonomic groups, among which the most important role is fulfilled by representatives of the order Myoida [73], to which the family Corbulidae is assigned [50], [74], [75]. Moreover, Botton and Ropes [61] demonstrated that bivalves of the genus *Corbula* formed part of the diet of *L. polyphemus*. It is therefore possible that the unusually high numbers of *Corbulomima* sp. at Owadów-Brzezinki may have constituted a kind of food attractor for *L. darwini* and as such, an important part of their diet. It is also important to realise that the majority of specimens of *Corbulomima* sp. are small (shell length between 3 and 12 mm), i.e. closely comparable with the size of bivalves preferred by the young and subadult representatives of extant horseshoe crabs [60], [61], [63], [64]. This may indicate that the depositional area of unit III could have been both nursery ground and feeding zone for *L. darwini* sp. nov.

Interestingly, the areas inhabited by modern horseshoe crabs (for details, see [5], [66], [70]) largely coincide with three biogeographical regions of current expansion of the family Corbulidae (i.e. Panamic-Caribbean-western Atlantic; eastern Atlantic-Mediterranean; and Indo West Pacific-Japan-Australia; [50]).

### Wide feeding spectrum and euryhalinity as the key to evolutionary success

Evolutionary changes which are hardly noticeable or show a very slow pace over long geological periods are generally defined as bradytely [76], [77]. The opposite process, i.e. the clearly evolutionary changes with very fast rate, is defined as tachytely. Simpson [76], [78] introduced the term bradytelic evolvers for groups that survived until today and show relatively little change since the very remote time when they first appeared in the fossil record. Moreover, Simpson established for each category a range centered around an average “lifetime” (for bradytelic species about 100 Ma). So defined, bradytely is nearly identical with the concept of “arrested evolution” proposed by Ruedemann [79], [80], and hipobradytely pertains to the exceptionally low rate of evolutionary change exhibited by cyanobacterial taxa proposed by Schopf [81]. The terms and their interpretations presented below (i.e. stabilomorphism; variomorphism) should be construed as detailed explanation of both these evolutionary processes. Due to the nature of the issues discussed, the average rate of evolutionary changes (i.e. horotely) is not included in the discussion, and will be presented in details elsewhere.

Modern Limulacea are classified as opportunistic feeders, which are able to live and reproduce in exceptionally diverse environments [82]. Each of the three genera - *Limulus* (1), *Tachypleus* (2) and *Carcinoscorpius* (3), occurs in moderately deep or shallow water zones along the east coast of North America (1) and south-east coast of Asia (2, 3). All three genera (four species in total) exhibit a high tolerance to changes of salinity in inhabited areas [66], [70], [82]. The Asian species, *C. rotundicaudata* is also observed to migrate considerable distances from the mouth to the source area of Hooghly River (i.e. up to 150 kilometers - compare [83], [84]). Attempts at interpretation of the vital environment of Mesozoic Xiphosurida indicate the potential existence of substantial saline tolerance in various genera, which potentially could be closely related to the present-day Limulacea [7], [20], [31], [85]–[87].

Levels of polymorphism and heterozygosity for allozyme loci in *Limulus* are comparable to those found in much more rapidly evolving organisms [88]. Generally, genetic variation of intra- and interspecific-populations of extant Limulacea is generally considered to be similar to the mean estimates for many other animals [89]. Intraspecific morphological variations are widely recognizable, but limited mainly to the size of exoskeletons and arising from this more or less noticeable differences in the proportions of the various morphological elements (compare [66], [67], [90]–[94]). The latter observation may indicate that impact of phenotypic plasticity on morphological modifications amongst horseshoe crabs of the genus *Limulus* is low.

On this basis, it may be assumed that the relative evolutionary conservatism noticeable in *Limulus*, and presumably amongst all known representatives of Xiphosurida, may be related to a unique formula of adaptative strategy, which is a combination of wide feeding spectrum, euryhalinity and eurythermality (*Limulus* survives in the very cold waters of Maine to the very warm waters of the shallow water coasts in Florida and Mexico - approximately 1°C to 30°C). In the case of *Limulus*, the adaptive properties discussed here could potentially provide the exceptionally long-term evolutionary success without significant morphological modification in respect to periodic fluctuations of the environment.

In order to systematize this phenomenon, we propose the term **stabilomorphism**, which may be understood as: *relative morphological stability of organisms in time and spatial distribution*, *the taxonomic status of which does not exceed genus level*. The definition refers exclusively to genera that have survived at least one of the great mass extinctions [95], or global biotic crises [96], and occur in contemporary environments. This means that the morphological structure of the conceptual stabilomorph has been virtually unchanged for more than 65 million years (e.g. *Limulus*), or they are known from the fossil record over comparable period of time (e.g. ammonoid genus *Phylloceras* [97]). The restricted applicability of this phenomenon may indicate, or even confirm, the multidimensionality of evolutionary processes of living organisms as well as their diversified pace, which presumably depend upon the type and quality of inherited adaptive strategy. It is also obvious that genetic factors, which may have an effect on the evolutionary success of known stabilomorphs, vary in individual cases.

**Figure 4:**
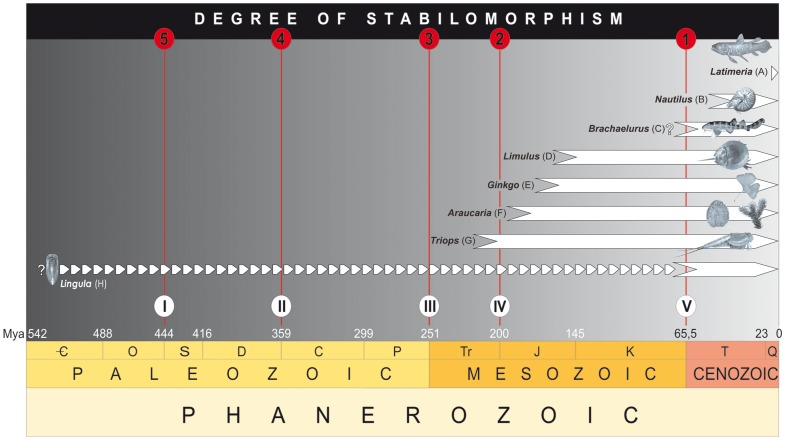
Graphical expression of the concept of stabilomorphism. Evident (D–G) and potential (C and H) stabilomorphs (i.e. organisms not extending the genus level, with very slow rate of evolutionary speciation). The proposed degree of stabilomorphism (1–5) is consistent with a five-step scale of the greatest mass extinctions in the history of life (I–V) (*sensu* [95]). Note that two genera (A and B), previously recognized as a model example of living fossils would thereby lose their present status (see explanations in text). Information about the stratigraphic ranges of stabilomorphs were taken from: (A) – [116]; (B) – [117]; (C) – [118]; (D) – data presented here; (E) – [119]; (F) – [120] and [121]; (G) – [122]; (H) – [123].

This notion demands precise confirmation of evolutionary longevity to regard certain genera as stabilomorphs. In order to classify the effectiveness of their adaptive strategy a five-step scale has been proposed. It's scope is related with the ability to survive considerable biotic crises that coincide with the greatest mass extinctions in the history of life ( = Big Five *sensu* [95] – Figure 4).

Genera other than *Limulus*, persisting not less than the above proposed time limit (i.e. at least 65 million years), and well known both contemporarily and from the fossil record, are commonly referred as living fossils [98], [99]. These genera (e.g. *Triops* [100], *Araucaria* [101], *Ginkgo* [102] and, probably, *Lingula* [103] and *Brachaelurus* [104]) seemto fulfil the criteria of stabilomorphs (Figure 4). Finally, in strict scientific terms, we propose that the imprecise and unusually broad popular-science concept of living fossil should be replaced by the easy-to-define term **stabilomorph**. In view of the hypothesis adopted here, some genera previously recognized as a model examples of living fossils (e.g. *Nautilus* [105], *Latimeria* [106] – Figure 4) lose their present status.

A variability of lesser features within a population, and, perhaps more visibly between populations over a period of time is a fundamental feature of all living organisms; this is the basis for acceptance of evolution as a process. The most important clue derived from a comparison of recent and fossil late-Jurassic *Limulus* species is that their level of adaptation, the quality of their adaptive strategy is so high (so effective), that small changes which had to continually occur over several millions years (in the case of *L. darwini*, at least 148 Ma) did not result in any significant morphology variations. In applying this concept to other supposed stabilomorphs, it implies that their adaptation reached such a point in their development, that they (as a group) could afford to “reject” any further changes.

One still open question is of primary or secondary origin of the unique features of hemolymph of recent Limulacea (for discuss, e.g. [107]), i.e. the violent amebocyte reaction to the presence of pirogenic endotoxins (i.e. lipopolysaccharides) produced by bacteria. This property is used, *inter alia*, in the medical industry, i.e. in the LAL test ( = Limulus Amebocyte Lysate) [108]–[110]. Presumably this feature increases the quality of the adaptive strategy within extant horseshoe crabs and, in consequence, affects the increased survival rate in subsequent populations [111], [112]. Naturally, issues of occurrences of this unique hemolymph feature among extinct Limulacea are highly problematic and it is regrettable that there is no opportunity to confirm this phenomenon within the fossil record.

Interestingly, specific counterparts of antibacterial properties of recent horseshoe crab hemolymph (e.g. anti-bacterial skin glands secretions) have also been recognized among representatives of other animal groups, characterized by strong evolutionary conservatism (e.g. frogs, crocodiles, sharks; for details, see – [113]–[115]).

However, it is important to recognise in the Author's hypothesis adopted here, some organisms with adaptive abilities opposite to stabilomorphs, exhibiting deficiency of morphological stability, featuring a number of phenotypic variants in response to environmental changes. They would have fossil record rather short in the evolutionary time scale. Organisms exhibiting these characteristics might be specified as **variomorphs**.

## Materials and Methods

The studied fossils of *Limulus darwini* were collected by authors during fieldwork in the Owadów-Brzezinki Quarry (lat. 51.374238°, lon. 20.136343°) in 2009–2013 (permission number: 1/2007/227, issued by Robert Siuda, Managing Director of Owadów-Brzezinki Quarry of the Nordkalk Company - branch in Poland). The collected material (both holotype and paratypes) are housed at the Institute of Paleobiology, Polish Academy of Science in Warsaw (ZPAL X.1/O-B). All studied specimens were on loan to the authors for the entire scientific study period. Fossils were measured using vernier callipers with an accuracy of 0.05 mm. All specimens have been prepared manually at the Museum of Association of Friends of Geosciences in Łódź and Institute of Paleobiology, Polish Academy of Science in Warsaw. The photography was carried out both in Łódź (Museum of Association of Friends of Geosciences) and Warsaw (Institute of Paleobiology, Polish Academy of Science). Photographs were taken using a Canon EOS 400D Digital Camera. All figures have subsequently been edited with Adobe Photoshop CS3 imaging software.

### Nomenclatural Act

The electronic edition of this article conforms to the requirements of the amended International Code of Zoological Nomenclature, and hence the new names contained herein are available under that Code from the electronic edition of this article. This published work and the nomenclatural acts it contains have been registered in ZooBank, the online registration system for the ICZN. The ZooBank LSIDs (Life Science Identifiers) can be resolved and the associated information viewed through any standard web browser by appending the LSID to the prefix “http://zoobank.org/”. The LSID for this publication is: urn:lsid:zoobank.org:pub: 8BB366FF-7999-4768-A0A5-72521D9606CB. The electronic edition of this work was published in a journal with an ISSN, and has been archived and is available from the following digital repositories: PubMed Central, LOCKSS (www.lockss.org).
